# Links Between the Neurobiology of Oxytocin and Human Musicality

**DOI:** 10.3389/fnhum.2020.00350

**Published:** 2020-08-26

**Authors:** Alan R. Harvey

**Affiliations:** School of Human Sciences, The University of Western Australia, Perron Institute for Neurological and Translational Science, Perth, WA, Australia

**Keywords:** oxytocin, music, dance, reward, empathy, trust, therapy

## Abstract

The human species possesses two complementary, yet distinct, universal communication systems—language and music. Functional imaging studies have revealed that some core elements of these two systems are processed in closely related brain regions, but there are also clear differences in brain circuitry that likely underlie differences in functionality. Music affects many aspects of human behavior, especially in encouraging prosocial interactions and promoting trust and cooperation within groups of culturally compatible but not necessarily genetically related individuals. Music, presumably *via* its impact on the limbic system, is also rewarding and motivating, and music can facilitate aspects of learning and memory. In this review these special characteristics of music are considered in light of recent research on the neuroscience of the peptide oxytocin, a hormone that has both peripheral and central actions, that plays a role in many complex human behaviors, and whose expression has recently been reported to be affected by music-related activities. I will first briefly discuss what is currently known about the peptide’s physiological actions on neurons and its interactions with other neuromodulator systems, then summarize recent advances in our knowledge of the distribution of oxytocin and its receptor (OXTR) in the human brain. Next, the complex links between oxytocin and various social behaviors in humans are considered. First, how endogenous oxytocin levels relate to individual personality traits, and then how exogenous, intranasal application of oxytocin affects behaviors such as trust, empathy, reciprocity, group conformity, anxiety, and overall social decision making under different environmental conditions. It is argued that many of these characteristics of oxytocin biology closely mirror the diverse effects that music has on human cognition and emotion, providing a link to the important role music has played throughout human evolutionary history and helping to explain why music remains a special prosocial human asset. Finally, it is suggested that there is a potential synergy in combining oxytocin- and music-based strategies to improve general health and aid in the treatment of various neurological dysfunctions.

## Introduction

The human species has evolved two universal systems of inter-personal communication, language, and music. These communication streams possess some common elements, for example, a requirement for processing certain aspects of pitch, rhythm, and syntax; however, there are also well-established differences in neural circuitry that are linked to differences in functionality. The possible evolutionary origin of musical behaviors in our species has been discussed elsewhere (e.g., Brown, 2000; Mithen, 2005; Fitch, 2006; Patel, 2008; Morley, 2013; Richter and Ostovar, 2016; Harvey, 2017) and is not considered in detail here. Language plays an essential role in cognition; it is the primary means by which modern humans communicate thoughts and ideas, it facilitates the sharing of learned information and knowledge within *and between* generations, it permits intuitive reasoning, foresight, and planning, and it likely co-evolved with our capacity to imagine times and places not personally experienced in our lifetime (Harvey, 2017). Language and the emergence and continued development of human culture seem to be closely intertwined, but then why do we also communicate and enjoy music and its partner dance? Why does music continue as a human universal and what is its significance to the species?

Music affects many aspects of human behavior, behaviors that may have had (and still have) adaptive benefits that presumably contribute to the ongoing existence of musicality in humans (e.g., Cross, 2009; Harvey, 2018). These benefits, which are by no means mutually exclusive, are thought to include the attraction and selection of mates, the facilitation of attachment between caregivers and preverbal infants, aiding the development of perceptual, cognitive and motor skills, and encouraging trust, social bonding, and mutual cooperation. In a group context music-related activities, including dance (Laland et al., 2016; Richter and Ostovar, 2016), encourage the formation of bigger social networks, help to define cultural identity, and may represent a “safe haven” in which individuals can interact and share experiences without revealing their innermost thoughts and fears. Evidence supporting the important role that music plays in promoting the development and maintenance of cooperative, prosocial behaviors comes from an increasing number of studies in children and in adults (Freeman, 2000; Kirschner and Tomasello, 2009; Tarr et al., 2014; Pearce et al., 2015; Schellenberg et al., 2015). Music, *via* its impact on various regions within the limbic system, is also rewarding, motivating, and facilitates aspects of learning and memory (Zatorre and Salimpoor, 2013; Koelsch, 2018). Lastly, and no less important, it is increasingly appreciated that musical activities are useful therapeutic tools, aiding in the treatment of some developmental disorders (Quintin, 2019), and capable of ameliorating behavioral and psychological symptoms in several neurodegenerative conditions (e.g., Abraha et al., 2017; Zhang et al., 2017; Särkämö and Sihvonen, 2018; Groussard et al., 2019; Pereira et al., 2019).

In this review article, these special characteristics of music are considered in light of recent research on the neurobiology of the peptide oxytocin. Oxytocin is a hormone, synthesized in the hypothalamus that has both peripheral and central actions. Peripherally, oxytocin has important roles before and after childbirth, acting on the uterus during labor and stimulating lactation. Centrally, oxytocinergic systems are thought to influence many complex human social behaviors including, for example, pair bonding, attachment and social memory, emotional empathy, trust and generosity, and suppression of anxiety. The first part of the review focuses on what is currently known about the physiological actions of oxytocin on cells in the mammalian central nervous system (CNS) and the peptide’s interactions with other neuromodulator systems including the closely related pituitary hormone arginine vasopressin (AVP), the stress-related hormone cortisol, and neurotransmitters such as dopamine and serotonin. The second section summarizes recent advances in our knowledge of the distribution of the peptide and its receptor in the human brain, the relationship between endogenous oxytocin levels and complex behavioral traits typical of *Homo sapiens*, and then reviews the diverse effects of intranasal oxytocin administration on human behavior. The final section discusses links between music-related activities and oxytocin expression, documenting the similarities between the generally prosocial behaviors engendered by oxytocin and the many positive effects that music has on human cognition, memory, and mental health. Oxytocin and music can also have beneficial effects on cardiovascular and immune systems, and it is argued that a better understanding of the multiple actions of the oxytocinergic system may lead to its synergistic use with music in a range of therapeutic applications in psychology and neurology.

## The Neuroscience of Oxytocin—Animal Studies

Oxytocin is a nine amino-acid peptide that is enzymatically derived from a larger peptide precursor made from the oxytocin gene. This peptide, or closely related versions of it, is involved in reproductive functions across almost all vertebrate species (Carter, 2014; Ebitz and Platt, 2014; Grinevich et al., 2016; Feldman, 2017; Jurek and Neumann, 2018) and its peripheral and central actions have been the subject of increasing interest in recent years (Jurek and Neumann, 2018)—as of 1st April 2020 there were more than 27,000 articles, including 3,700 reviews, listed on the NIH PubMed search engine.

In the mammalian brain, oxytocin is synthesized predominantly by magnocellular neurons in the supraoptic (SON) and paraventricular (PVN) nuclei of the hypothalamus, by some parvocellular neurons in PVN, and in accessory magnocellular nuclei of the hypothalamus. There is also some expression in peripheral tissues such as the gonads, kidney, and pancreas although the oxytocin generated there is unlikely to enter the CNS (Jirikowski, 2019). Many oxytocin-expressing neurons likely bifurcate, with a “traditional” endocrine-related projection to the posterior pituitary for systemic release into the bloodstream and a second central branch projecting to about 50 brain regions, including the sensory and prefrontal cortex, nucleus accumbens in the ventral striatum, amygdala, hippocampus, hypothalamus and ventral tegmentum (Grinevich and Stoop, 2018). In the circulation, oxytocin has a half-life of only a few minutes before it is metabolized in the liver and kidneys. This is an important point that will be returned to later in this review when discussing how best to measure and interpret peripheral oxytocin levels in humans.

In animal models, a variety of methods have been used to investigate the molecular and cellular properties of oxytocin and its receptor (OXTR), and to better understand the nature of the relationship between the physiology and pharmacology of oxytocin signaling and overt behavior (Jurek and Neumann, 2018; Mitre et al., 2018; Cilz et al., 2019; Neumann and Landgraf, 2019; Tan et al., 2019; Raam, 2020). These methods include neuroanatomical pathway tracing, immunohistochemistry, electron microscopy. receptor autoradiography, *in situ* hybridization, electrophysiology, microdialysis, and functional magnetic resonance imaging (fMRI). Experimental interventions have also been used to perturb the oxytocinergic system such as intracerebral infusion of the peptide, the use of receptor agonists or antagonists, antisense methods, optogenetic and chemogenetic stimulation of oxytocinergic neurons, conditional deletion of OXTR, and the use of genetically engineered reporter mice.

### The Oxytocin Receptor

Oxytocin binds with high affinity to its specific receptor OXTR and can initiate an array of intracellular signaling cascades and transcriptional events (Chatterjee et al., 2016; Busnelli and Chini, 2017; Jurek and Neumann, 2018). There is also significant crosstalk with structurally related AVP receptors, most particularly the AVP1a receptor (AVPR1a; Bakos et al., 2018; Grinevich and Stoop, 2018; Song and Albers, 2018). In turn, AVP can also bind to OXTR, however, the specificity of action largely remains, probably due to differences in the distribution of oxytocin vs. AVP containing axons (Grinevich and Stoop, 2018; Rogers et al., 2018; Song and Albers, 2018; Pekarek et al., 2020). Nonetheless, there are potential sites of interaction in some brain regions (Smith et al., 2019), and there is evidence of functionally relevant spillover of oxytocin into the extracellular space beyond traditional synaptic sites (Busnelli and Chini, 2017; Chini et al., 2017; Song and Albers, 2018). OXTRs are widely distributed and found in many neuronal types, expressed on cell bodies, dendrites, and axon terminals, and the receptor is also expressed by astrocytes (Wang et al., 2017; Bakos et al., 2018; Young and Song, 2020). In different species, the receptor seems to be specifically enriched in those sensory/perceptual systems that are most relevant to conspecific maternal as well as more general socially interactive behaviors (Grinevich and Stoop, 2018; Pekarek et al., 2020).

### The Physiology of Oxytocin

In the CNS, oxytocin can affect various ion channels, increase intracellular calcium ion concentrations, alter membrane excitability and enhance long-term potentiation (LTP) in neurons (Tomizawa et al., 2003; Lee et al., 2015; Lin and Hsu, 2018; Tirko et al., 2018). Oxytocin signaling also increases the expression of neurotrophic factors such as brain-derived neurotrophic factor (BDNF; Bakos et al., 2018; Zhang et al., 2020), of relevance to later discussion focussed on oxytocin, social learning/memory, and hippocampal function. The peptide can also act presynaptically to affect neurotransmitter secretion (Dölen et al., 2013; Bakos et al., 2018). Overall, from a physiological perspective, oxytocin influences cell viability, synaptic and structural plasticity in neurons (Bakos et al., 2018; Jurek and Neumann, 2018; Pekarek et al., 2020), and modulates the balance of excitatory and inhibitory activity in regions such as the cerebral cortex and hippocampus (e.g., Mitre et al., 2016; Grinevich and Stoop, 2018; Lin and Hsu, 2018; Lopatina et al., 2018; Tirko et al., 2018; Cilz et al., 2019; Maniezzi et al., 2019; Tan et al., 2019), amygdala (Crane et al., 2020), and nucleus accumbens (Moaddab et al., 2015; Cox et al., 2017). Rodents lacking oxytocin or OXTR display impaired sociability and social memory (Ferguson et al., 2000). and conditional deletion of OXTR in the hippocampus negatively affects LTP and impairs long-term social recognition memory (Lin et al., 2018).

Likely increasing its diversity of action, oxytocin also interacts with several other receptors and neuromodulatory systems. For example, the peptide: (i) potentiates excitatory dopamine-mediated synaptic transmission (Li et al., 2020); (ii) interacts with a class of serotonin receptor (Chruścicka et al., 2019) and affects serotonin release (Yoshida et al., 2009); (iii) modulates signaling mediated by opioid receptors (dal Monte et al., 2017; Meguro et al., 2018; Salighedar et al., 2019); and (iv) activates TRPV2 channels (Van den Burg et al., 2015). There are also dynamic interactions with steroids (Jirikowski et al., 2018) and oxytocin levels are negatively correlated with cortisol, significantly modifying responses to stress (Lee et al., 2015; Schladt et al., 2017; Latt et al., 2018; Masis-Calvo et al., 2018; Neumann and Landgraf, 2019).

The foregoing section has, of necessity, over-simplified the physiological effects of oxytocin on neural tissue in animals, and more in-depth reviews are available (e.g., Bakos et al., 2018; Jurek and Neumann, 2018; Mitre et al., 2018; Neumann and Landgraf, 2019). However, some discussion of animal-based research is warranted because, given that the peptide is highly conserved in evolution it is likely that similar wide-ranging molecular and cellular mechanisms are operative in the human brain (see also Grinevich and Neumann, 2020). From a behavioral perspective, animal studies reveal that oxytocin has an important role in pair-bonding and maternal attachment, in moderating affiliative behaviors and conspecific social recognition, and in modulating the formation and maintenance of episodic memories, whether they be positive or negative. The next section will show that oxytocin has generally similar effects on human social behavior, but these effects would seem to be more subtle and complex in cognitively advanced members of *Homo sapiens*, extending to personality traits, emotional empathy, trust, altruism, reciprocity, group conformity, social decision making and so on.

## Oxytocin in Humans

### Oxytocinergic Networks

In humans, immunoreactive oxytocinergic fibers are sparse but present in all cortical layers of the orbitofrontal cortex and anterior cingulate (Rogers et al., 2018). The fibers were found to have large varicosities usually associated with *en passant* boutons—likely sites of oxytocin release into the surrounding neuropil (Busnelli and Chini, 2017; Chini et al., 2017; Song and Albers, 2018). Fibers immunoreactive for AVP were also seen in these cortical regions and in the insular and olfactory cortices. Using antibodies to the receptor, OXTR was first identified in parts of the amygdala, anterior cingulate cortex, hypothalamus, and preoptic area, olfactory nucleus, and some brainstem nuclei (Boccia et al., 2013). A more recent extensive survey of the oxytocin system analyzed the distribution of the gene encoding OXTR as well as the gene encoding the oxytocin prepropeptide and the gene encoding CD38, a transmembrane protein needed for oxytocin secretion (Quintana et al., 2019). OXTR gene expression was widespread throughout the brain, significantly higher in olfactory bulbs, but also higher in the caudate, putamen, pallidum, and hypothalamus; levels were also greater than average in the hippocampus, parahippocampal region, amygdala, parts of the temporal lobe and anterior cingulate cortex. Expression of the gene was “reproducible, regardless of individual differences, such as ethnicity and sex” (Quintana et al., 2019).

The pattern of expression was essentially similar for the CD38 gene, with significantly increased expression in caudate, putamen, pallidum, thalamus, and anterior cingulum. For both genes, expression was significantly lower in the cerebellum. Interestingly, there was co-expression with several genes involved in dopaminergic and muscarinic cholinergic signaling, suggesting potential pathway interactions perhaps similar to those suggested for the opioids (dal Monte et al., 2017). Co-expression with genes involved in the regulation of metabolism and appetite was also seen. According to Quintana et al. (2019), “the oxytocin pathway gene maps correspond with the processing of anticipatory, appetitive, and aversive cognitive states.” Interaction with dopaminergic and cholinergic systems is likely to add to the broad impact of oxytocin on social behaviors, motivation, reward, desire, anxiety, and the processing of emotions.

### Receptor Polymorphisms and Behavior

In children, adolescents, and adults, genetic variants of the OXTR gene are linked to an individual’s response to stress (Rodrigues et al., 2009) and altered prosocial/affiliative behaviors and empathy. The need for pleasant social company is increased after a stressful event, a need that varies depending on which alleles of OXTR are present (Sicorello et al., 2020). Anatomically, there are subtle changes in structure and inter-connectivity of hypothalamus and parts of the limbic system, and mutations have been implicated in a range of highly maladaptive, sometimes psychopathic traits (e.g., Israel et al., 2008; Tost et al., 2010; Dadds et al., 2014; Aspé-Sánchez et al., 2016; Feldman et al., 2016; Gedeon et al., 2019; Poore and Waldman, 2020). A recent neuroimaging study examining the effect of OXTR alleles on resting-state networks reported that receptor genotype affected connectivity between the right hippocampus, medial prefrontal cortex, dorsal anterior cingulate cortex, amygdala, basal ganglia and thalamus (Luo et al., 2020). The functional impact that alleles of OXTR have on social behavior is however complex and not always consistent across studies, and is affected by factors such as gender, age, upbringing, and culture (Tost et al., 2010; Feldman et al., 2016; Fujiwara et al., 2019; Plasencia et al., 2019; Poore and Waldman, 2020). Environmental epigenetic influences on the OXTR function that influence social interactions must also be considered (Chen et al., 2020), and as described earlier there may be differential interactions with other neuromodulatory systems such as AVP, the opioids, steroids, and various catecholamines.

### Measurement of Endogenous Oxytocin

As yet it has not proved possible to measure oxytocin levels in the living human brain, thus endogenous oxytocin measurements are obtained from either plasma, saliva, or urine. Interpretation of these peripheral measures of oxytocin is however difficult for several reasons (Ebstein et al., 2012; Leng and Ludwig, 2016; Mitre et al., 2016; Valstad et al., 2017; Jurek and Neumann, 2018). First, peripheral oxytocin levels are related to the release of the peptide from the posterior pituitary and do not necessarily reflect levels of the peptide within specific regions of the brain that contain neurons expressing OXTR. Second, even when undertaking peripheral measurements, compared to saliva there is, in animals at least, a more consistent relationship between blood plasma levels of oxytocin and levels of the peptide found in cerebrospinal fluid (Valstad et al., 2017). Third, as pointed out by Jurek and Neumann (2018): “basal plasma or brain oxytocin levels might strongly depend on individual events occurring within the last hour(s) before sampling (e.g., fear of hospital or laboratory, prior eating, rushing to the laboratory, or sex) or on the time of the day.” And all is compounded by the fact that circulating levels of oxytocin are normally low, even exogenous peptide is rapidly eliminated 1–2 h after intranasal delivery, and to measure native (unbound) oxytocin levels requires sophisticated techniques for specificity and accuracy of analysis (Franke et al., 2019, 2020). Nonetheless, and given these caveats, some intriguing and important observations have come from peripheral endogenous oxytocin measurements in humans.

### Nurturing and Bonding

Plasma and/or salivary oxytocin levels rise postpartum when mothers interact and bond with their infants (Matthiesen et al., 2001; Feldman et al., 2007; Feldman, 2012; Gordon et al., 2010). These interactive behaviors include gaze, facial expression, vocalizing using the preverbal maternal-infant communication known as “motherese,” affectionate touch, and so on (Kerr et al., 2019). The increase in serum oxytocin when mothers interact and bond with their own smiling/happy infants is higher in mothers rated as having “secure” attachment to their offspring (Strathearn et al., 2009) and with a sensitive temperament (Strathearn et al., 2012). In these mothers, fMRI revealed greater activity in the hypothalamus/pituitary region and in reward centers in the ventral striatum. The rise in oxytocin is, at least in part, related to the amount of maternal gaze directed towards the child (Kim et al., 2014). Intranasal oxytocin also increases a father’s neural response to images of their young children, with increased activity in caudate, anterior cingulate, and visual cortex (Li et al., 2017a).

Lullabies are a universal way of soothing infants (Mehr et al., 2018), and it is thus of interest that vocalization by mothers increases levels of salivary oxytocin (and reduces cortisol) in their children, although admittedly these were older girls aged between 7 and 12 years old (Seltzer et al., 2010). In comparing maternal vs. paternal changes in endogenous oxytocin during early parent-infant bonding, both mothers and fathers showed increases but there were dimorphic differences that depended on the type of interaction (Gordon et al., 2010). The development of affiliative behaviors between caregiver and infant, linked especially to oxytocin, leads to plasticity and adaptations in both the parent and infant (Feldman, 2015). Remarkably, the basal level of oxytocin measured in the saliva, and certain polymorphisms in the OXTR gene, are transgenerationally associated with the type of parental care that is given, influencing affiliative and social behaviors across as many as three generations within a family (Fujiwara et al., 2019).

### Oxytocin in Adolescents and Adults

Endogenous plasma oxytocin concentrations vary with age and there are differences between males and females, young women having the highest and old men the lowest levels (Plasencia et al., 2019). Experimentally, levels of salivary oxytocin were higher when female subjects were confronted with a novel situation, associated with reduced stress and greater trust, compared with a later familiarization session (Tops et al., 2013). Higher levels of plasma oxytocin in women but not men were linked to indicators of relationship stress and attachment anxiety (Taylor et al., 2010; Weisman et al., 2013; Moons et al., 2014). In young males, lower urinary oxytocin (but not AVP) levels were linked to lower measures of empathy and trust, presumably associated with a greater propensity for aggressive behavior (Malik et al., 2012; Weisman et al., 2013; de Jong and Neumann, 2018; Berends et al., 2019), and in males social cognitive ability was correlated with plasma oxytocin concentrations (Deuse et al., 2019; Strauss et al., 2019).

### Effects of Exogenous Administration of Oxytocin

Exogenous delivery of oxytocin affects neural processing and has consistently been reported to influence a wide range of interactive human behaviors. These behaviors have been described in different ways and using different terminologies. They include pair bonding, attachment, and social learning/memory, social salience and emotional empathy, recognition and interpretation of emotions, behavioral synchrony, familiarization and within group co-operation, altruism, generosity and trust, reward sensitivity, calmness and reduction of stress, and amelioration of anxiety (anxiolytic effects; e.g., Kosfeld et al., 2005; Baumgartner et al., 2008; Ditzen et al., 2009; Strathearn et al., 2009; Hurlemann et al., 2010; De Dreu, 2012; Fischer-Shofty et al., 2012; Tops et al., 2013; Bethlehem et al., 2014; Preckel et al., 2014; Shamay-Tsoory and Abu-Akel, 2016; Feldman, 2017; Fineberg and Ross, 2017; Leppanen et al., 2017; Wang et al., 2017; Ellenbogen, 2018; Geng et al., 2018; Jurek and Neumann, 2018; Rilling et al., 2018; Alos-Ferrer and Farolfi, 2019; Liu et al., 2019; Tillman et al., 2019; Sicorello et al., 2020; Wu et al., 2020). Analyses of the impact of oxytocin on neural activity imaged in the brains of healthy subjects generally reflect this, with altered activity in interconnected structures associated with valence, salience, trust, prosocial behavior and mentalizing, including the amygdala, insula, nucleus accumbens, lateral septum, anterior cingulate, hippocampus, caudate, tempero-parietal cortex, dorsomedial and dorsolateral prefrontal cortex (e.g., Kirsch et al., 2005; Rilling and Sanfey, 2011; Bethlehem et al., 2013; Eckstein et al., 2017; Wang et al., 2017; Rilling et al., 2018; Kumar et al., 2020; Wu et al., 2020).

The great majority of studies emphasize positive, prosocial behavioral outcomes after exogenous oxytocin administration; however, it is important to emphasize that not all studies describe these effects (Keech et al., 2018; Tabak et al., 2019; Erdozain and Peñagarikano, 2020) and some antisocial outcomes have been reported, including increased competitive and aggressive tendencies, particularly in males (Fischer-Shofty et al., 2012; Alcorn et al., 2015; Ne’eman et al., 2016; de Jong and Neumann, 2018; Gedeon et al., 2019). Others have also reported differential effects of exogenous oxytocin on women compared to men (e.g., Rilling et al., 2012, 2018; Preckel et al., 2014; Feng et al., 2015; Chen et al., 2016; Bredewold and Veenema, 2018; Bartz et al., 2019; Xu et al., 2020).

Overall, variation in the effects of administered oxytocin is linked to: (i) gender differences; (ii) whether individuals possess intrinsic pro- or antisocial personality traits—sometimes related to polymorphisms in, or the methylation state of, OXTR; (iii) early environmental experience; and/or (iv) the social cues and psychological context when testing is undertaken (e.g., Guastella and MacLeod, 2012; Evans et al., 2014; Nishina et al., 2015; Chen et al., 2016; Feldman et al., 2016; Lambert et al., 2017; Aydogan et al., 2018; de Jong and Neumann, 2018; Wagner and Echterhoff, 2018; Fragkaki and Cima, 2019; Gedeon et al., 2019; Liu et al., 2019; Sicorello et al., 2020). As mentioned earlier, oxytocin and AVP can activate each other’s receptors, although the differential distribution of fibers and receptors may limit crosstalk (Rogers et al., 2018; Song and Albers, 2018). There may be little interaction under normal conditions of endogenous release, but perhaps there is more crosstalk after intranasal application of higher concentrations of oxytocin which may contribute to some of the complexity of behavioral outcomes.

## The Links Between Oxytocin and Music

**Figure 1:**
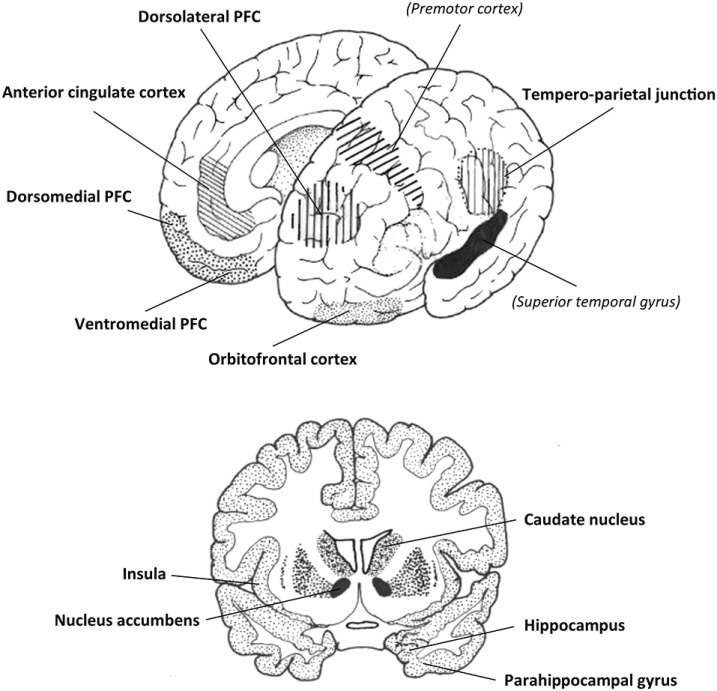
Two schematic views of the brain showing many of the regions in common that are reported to be involved in: (i) processing subjective/arousing/rewarding/emotional aspects of human musicality; (ii) performing or responding to a range of interactive social tasks; and (iii) oxytocinergic processing. Two other potentially relevant regions are shown in italics. Amygdala not shown. PFC, the prefrontal cortex. Both diagrams modified from Harvey (2017).

Oxytocin is an ancient peptide, in mammals universally involved in reproductive biology, modulating social learning and affiliative behaviors, as well as modifying responses to adverse conditions (Ebitz and Platt, 2014; Feldman et al., 2016; de Jong and Neumann, 2018). In *Homo sapiens*, it is conceivable that the unique prosocial, harmonizing activities of music and dance incorporated, perhaps even required, elements of this pre-existing oxytocinergic network. Music encourages affiliative interactions in infancy and adulthood, aids in the development of perceptual, cognitive, and motor skills, promotes trust and reduces a sense of social vulnerability, is rewarding and motivating, and has a beneficial effect on aspects of learning and memory. Music and its evolutionary partner dance (Richter and Ostovar, 2016) also promote synchrony and social interaction, contribute to cultural identity, and encourage the formation of cooperative networks. Based on the experimental work described above, it should be apparent that many of these musical influences on human behavior are also characteristic of many of the psychological and sociological effects of oxytocin. These associations become even clearer when comparing the neural networks that are: (i) activated when listening to music perceived as being rewarding and pleasurable with; (ii) regions that process behaviors that involve social cooperation, empathy and altruism; and (iii) the distribution of oxytocinergic fibers and OXTR in the human brain (Figure 1).

### Music Networks

As described in detail elsewhere (Harvey, 2017), and acknowledging that overlap in activity maps does not, *a priori*, mean that the same circuits are involved (Peretz et al., 2015), some of the basic elements of language and music such as pitch and rhythm share similar neural substrates, but clearer differences become apparent when more extended processing networks are considered. In right-handers at least, there is a left hemisphere bias for language and speech while the right hemisphere is biased more for music, and separate processing areas specific for these two communication streams have now been identified within secondary auditory regions in the superior temporal gyrus (Angulo-Perkins et al., 2014; Norman-Haignere et al., 2015). Most relevant to the present discussion are the limbic pathways and multiple cortical regions known to be activated by the overall subjective experience and emotional impact of music (Koelsch, 2018).

The limbic system, which includes the hippocampus, parahippocampal gyrus, amygdala, and cingulate cortex, is involved in several functions including learning, memory, motivation and emotional responsiveness. Music can induce activity in all these regions, while music that is perceived as arousing and is appreciated also drives dopaminergic activity in nucleus accumbens in the ventral striatum, an anticipatory and reward center (Blood and Zatorre, 2001; Menon and Levitin, 2005; Boso et al., 2006; Salimpoor et al., 2011; Zatorre and Salimpoor, 2013; Mueller et al., 2015; Ferreri et al., 2019; Gold et al., 2019; Shany et al., 2019). Music that evokes strong emotional valence is associated with altered activity not only in the superior temporal gyrus but also in the caudate nucleus, insula, thalamus, cingulate cortex, orbitofrontal, dorsomedial, dorsolateral and ventromedial prefrontal cortex, inferior frontal cortex and supplementary motor area (e.g., Blood and Zatorre, 2001; Koelsch et al., 2006; Mitterschiffthaler et al., 2007; Chapin et al., 2010; Brattico et al., 2011; Pereira et al., 2011; Khant et al., 2012; Altenmüller et al., 2014; Koelsch, 2018; Särkämö and Sihvonen, 2018; Sachs et al., 2019). Of course, music involves more than just listening, and imaging of people—alone or with others—creating and improvising jazz, rap or rock music has also revealed increased neural activity in the medial frontal lobe and altered, usually decreased, activity in the dorsolateral prefrontal cortex when compared to the same subjects playing or singing “formulaic sequences” (Limb and Braun, 2008; Liu et al., 2012; Donnay et al., 2014; Tachibana et al., 2019).

### Behavioral Networks

Numerous studies have used fMRI to image healthy subjects performing and responding to interactive social tasks that may involve altruism, empathy, trust and cooperation, norm-abiding behavior, mentalizing, and/or an appreciation of nuanced social context (Figure 1). These studies generally find increased functional activity in the nucleus accumbens, amygdala, parahippocampal gyrus, caudate nucleus, insula, anterior cingulate cortex, superior temporal cortex, tempero-parietal junction, and several regions in the prefrontal cortex (medial orbitofrontal, medial prefrontal, ventromedial, dorsolateral, dorsomedial; e.g., O’Doherty, 2004; Völlm et al., 2006; Rilling et al., 2008; Cooper et al., 2010; Rilling and Sanfey, 2011; Rushworth et al., 2011; Carter et al., 2012; Korn et al., 2012; Fukuda et al., 2019).

### Oxytocinergic Systems

Oxytocin fibers have been identified in the orbitofrontal cortex and anterior cingulate cortex (Rogers et al., 2018), and there are high levels of the receptor in the olfactory bulb, amygdala, hippocampus, parahippocampal gyrus, regions in the temporal lobe, anterior cingulate cortex, hypothalamus and preoptic area, and some brainstem nuclei (Boccia et al., 2013; Quintana et al., 2019). Intranasal oxytocin administration alters neural activity in structures such as the amygdala, insula, nucleus accumbens, anterior cingulate cortex, hippocampus, caudate, tempero-parietal cortex, dorsomedial and dorsolateral prefrontal cortex (e.g., Kirsch et al., 2005; Lischke et al., 2012; Bethlehem et al., 2013; Eckstein et al., 2017; Wang et al., 2017; Rilling et al., 2018; Kumar et al., 2020; Wu et al., 2020). Finally, altered OXTR genotypes have been found to correlate with altered local network metrics and functional connectivity between the hippocampus, medial prefrontal cortex, dorsal anterior cingulate cortex, amygdala, basal ganglia and thalamus (Luo et al., 2020).

Clearly then, musicality, cooperative prosocial interactions, and the oxytocinergic system are linked to neural activity in several common regions and interconnected networks in the brain of modern humans; the most consistently involved components being the hippocampus, parahippocampal gyrus, amygdala and anterior cingulate cortex, the caudate nucleus and nucleus accumbens, insula, superior temporal gyrus and orbitofrontal, ventromedial, dorsomedial and dorsolateral prefrontal cortex (Figure 1). Numerous examples of the close interrelationship between oxytocin and prosocial human behaviors have been presented, promoting group empathy and the participation in collective decision making, all involving a shift from personal concerns to more communal interests, including a willingness to learn from others (Zak and Berroza, 2013; Shalvi and De Dreu, 2014; De Dreu and Kret, 2016; De Wilde et al., 2017; Ten Velden et al., 2017; Schiller et al., 2020; Xu et al., 2020). But to what extent does oxytocin provide a nexus between these behaviors and music? What impact does music have on peripheral oxytocin release and OXTR expressing networks in the human brain, and can exogenous oxytocin administration synergistically affect performance and responsiveness to music?

### Experimental Studies on Music and Oxytocin

Only a few studies have directly examined the impact of music on oxytocin expression, in solo or ensemble settings. As described earlier, comforting maternal vocalizations—which can have music-like properties—by themselves have been shown to increase oxytocin levels and reduce cortisol in young daughters (Seltzer et al., 2010). It was recently reported that salivary oxytocin levels are reduced in maltreated children (Suzuki et al., 2020) and it is therefore of interest that, following a program of group drumming sessions for “emotionally disturbed” children, salivary oxytocin concentrations were increased in both boys and girls, significantly so when comparing practice and free play sessions performed by boys aged 8–12 years (Yuhi et al., 2017). In adults, salivary oxytocin levels were also found to be raised after a singing lesson, amateur singers, in particular, expressing a heightened sense of well-being (Grape et al., 2003), and raised levels were also reported after choral singing (Kreutz, 2014). In one sensory study, it was found that listening to slow relaxing music was associated with raised salivary oxytocin levels and lower heart rate, whereas fast music had little impact on oxytocin but reduced cortisol levels and increased arousal (Ooishi et al., 2017). The effect of relaxing music on moderating salivary cortisol levels after the stress has also been noted (Khalfa et al., 2003).

The nature of the musical activity is important because an increase in plasma oxytocin levels in members of a vocal jazz group was only recorded when singers were improvising together (Keeler et al., 2015), likely due to altered activity in the prefrontal cortex and enhanced affiliative and prosocial interactions (Limb and Braun, 2008; Liu et al., 2012; Donnay et al., 2014; Tachibana et al., 2019). Schladt et al. (2017) reported that salivary oxytocin levels slightly increased when subjects were solo singing but were decreased when singing in a choir. In that same study, cortisol levels were reduced in both situations, but choral participants described greater feelings of happiness and reduced worry. Of course, performing music can be stressful, perhaps especially in a solo compared to an ensemble/choral situation. Indeed, the intranasal application of oxytocin has recently been shown to increase positive interactions between performers and reduce performance anxiety (Sabino et al., 2020). The reported variability in measured levels of oxytocin and markers of stress such as cortisol reflects the complex, and highly interactive, sexually dimorphic systems that are involved (Brown et al., 2016). The other issue that should be borne in mind, discussed in detail earlier, is that measurement of salivary or urinary oxytocin levels do not necessarily reflect the concentration of the peptide in OXTR expressing regions in the brain (Leng and Ludwig, 2016; Jurek and Neumann, 2018), although there is a closer relationship between plasma oxytocin levels and those in cerebrospinal fluid (Valstad et al., 2017).

Overall, whilst there is a clear trend for increased endogenous oxytocin and reduced cortisol in subjects involved in musical activities, more controlled trials are needed in this area because communal music experiences are prime examples of human social engagement. From a physiological and psychosocial perspective, group music-making such as choral singing increases connectedness, heightens empathy, reduces depression and improves mood, is arousing and stimulates cognition, and has systemic health benefits including improved immune competency, reduced cytokine and inflammatory markers, lowered blood pressure and reduced cortisol and ACTH levels (Kuhn, 2002; Khalfa et al., 2003; Kreutz et al., 2004; Dunbar et al., 2012; Fancourt et al., 2014; Keeler et al., 2015; Pearce et al., 2015; Stewart and Lonsdale, 2016; Johnson et al., 2017; Ooishi et al., 2017; Finn and Fancourt, 2018; Kang et al., 2018; Moss et al., 2018; Perkins et al., 2018; Walker et al., 2019). The impact of exogenous oxytocin is relevant here because of the positive effect that it has on individual stress levels and the promotion of group empathy, reciprocal trust and collective social decision making, all involving a shift from personal to group agency (Zak and Berroza, 2013; Chen et al., 2016; De Dreu and Kret, 2016; De Wilde et al., 2017; Ten Velden et al., 2017; Sicorello et al., 2020; Xu et al., 2020). Music and the community associated with it may be especially important to individuals who are lonely and/or who have lower emotional empathy and exhibit fewer prosocial traits (e.g., Berends et al., 2019; Fragkaki and Cima, 2019; Liu et al., 2019; Johnson et al., 2020; Schiller et al., 2020).

### Links to Other Neuromodulatory Systems

Rhythm in music induces bodily movement and akin to music, dance is a universal human behavior (Levitin et al., 2018). From an evolutionary perspective, it has been argued that dance advantages humans “by contributing to sexual reproduction signaling, cooperation, social bonding, infant care, violence avoidance as well as embodied individual and social communication and memorization” (Richter and Ostovar, 2016). To my knowledge, there have not, to date, been any substantive reports on how oxytocin levels are affected by solo or group dance. Yet the impact of dance, especially in a coordinated group context, on increased empathy (Gujing et al., 2019) social bonding (Tarr et al., 2015, 2016), cognitive performance, general fitness and well-being (Kattenstroth et al., 2010, 2013; Zilidou et al., 2018; Douka et al., 2019) is clear. Choral singing and dance have both been reported to increase pain threshold, viewed as a surrogate for levels of circulating β-endorphin (Dunbar et al., 2012; Tarr et al., 2015; Weinstein et al., 2016). This peptide binds to μ-opioid receptors and plays a role in social networking and maintaining social bonds (Pearce et al., 2017). These observations are important, but it should be noted that many other factors can influence the perception and processing of pain (Millan, 2002), including oxytocin (Gamal-Eltrabily et al., 2020; Hilfiger et al., 2020; Schneider et al., 2020), which as described earlier positively modulates signaling mediated by opioid receptors (dal Monte et al., 2017; Meguro et al., 2018; Salighedar et al., 2019). Furthermore, depending on age and health status, the perception of pain does not necessarily reflect circulating β-endorphin levels (Bruehl et al., 2017; Ahn et al., 2019).

AVP is also thought to influence human behavior in many ways (Neumann and Landgraf, 2012; Benarroch, 2013) although significant effects are not always evident (Tabak et al., 2019). AVP and oxytocin may interact with each other to influence prosocial vs. antisocial behaviors, trust vs. aggression, fear and so on (Huber et al., 2005; Veenema and Neumann, 2018; Ebstein et al., 2012; Rilling et al., 2012; Jurek and Neumann, 2018; Song and Albers, 2018; Berends et al., 2019), at least some of which are sexually dimorphic (Rilling et al., 2014; Feng et al., 2015; Bredewold and Veenema, 2018). Comparison of endogenous AVP and oxytocin levels in plasma from young and old men and women revealed a negative correlation between all groups, higher AVP levels associated with greater “attachment anxiety” (Plasencia et al., 2019) and pair-bond distress in men (Taylor et al., 2010). Polymorphisms in the AVPR1a receptor have been linked to variability in aggression, response to stress, trust, and altruistic behaviors (Israel et al., 2008; Moons et al., 2014; Aspé-Sánchez et al., 2016; Nishina et al., 2019). Whilst there is as yet no evidence that endogenous levels of circulating AVP are altered by musical activity, AVPR1a receptor polymorphisms have been linked to musical aptitude (Pulli et al., 2008; Ukkola et al., 2009; Liu et al., 2016; Mariath et al., 2017), memory (Granot et al., 2007, 2013) and appreciation (Ukkola-Vuoti et al., 2011), as well as music and dance creativity (Bachner-Melman et al., 2005; Israel et al., 2008; Oikkonen et al., 2016). On the other hand, receptor polymorphisms were not more common in choral singers compared with people designated as non-musicians (Morley et al., 2012).

### Anxiety, Extinction, and PTSD

Participation in music is rewarding; it encourages prosocial interactions, facilitates social cognition, and promotes cooperation within groups of culturally compatible but not necessarily genetically related individuals. Ensemble music-making, and communal choral and dance activities, involve synchronized and coordinated activity with the special attribute of allowing individuals to be subsumed within a greater, living whole. Perhaps most importantly, the generally ambiguous, non-propositional nature of music provides a safe, usually risk-free space where individual thoughts and emotions, personal autobiographical memories and ambitions, can exist in a cooperative and interactive social context. Participation in musical activities can help individuals who lack self-confidence, who lack trust and may feel socially excluded, reduces fear and a sense of vulnerability, and can diminish potential conflict: “Music allows participants to explore the prospective consequences of their actions and attitudes toward others within a temporal framework that promotes the alignment of participants’ sense of goals” (Cross, 2009).

This putative “safe haven” aspect of human musicality is similar to some of the behavioral effects elicited by oxytocin and further supports the proposed close links between music and oxytocinergic systems. Although not evident in all trials (Donadon et al., 2018), many studies have reported that exogenous delivery of oxytocin has anxiolytic and calming effects on human behavior (Neumann and Slattery, 2016; Wang et al., 2017; Lancaster et al., 2018; Yoon and Kim, 2020), enhancing the detection of threat (Lischke et al., 2012; Bredewold and Veenema, 2018) and facilitating the extinction of fearful or distressing memories (Kirsch et al., 2005; Hu et al., 2019; Koch et al., 2019; Triana-Del Río et al., 2019). Indeed, endogenous oxytocin levels are reduced in individuals with emotional trauma and in sufferers of posttraumatic stress disorder (PTSD; e.g., Frijling et al., 2015) and the administration of oxytocin may prove to be a useful therapeutic strategy (Giovanna et al., 2020). The acquisition and processing of autobiographical experiences, including fear and extinction, involves the hippocampus and ventromedial prefrontal cortex (Bonnici and Maguire, 2018; Dunsmoor et al., 2019) as well as interactions with the amygdala (Dunsmoor et al., 2019; Hasan et al., 2019). Activity in all these regions is associated with aspects of both musical and oxytocinergic processing. Concerning extinction, reducing the emotional impact of remembering fearful and threatening events involves substitution with novel, less impactful memories during the retrieval and reconsolidation process, a process facilitated by oxytocin (Hu et al., 2019; Triana-Del Río et al., 2019) and one that may also be aided by participation in the safe, neutral and motivating mental space evoked by communal music-related activities. In this context, music therapy has been suggested as a possible treatment for PTSD (Beck et al., 2018), and its use in association with oxytocin administration may prove even more beneficial.

Of the many endogenous opioids, β-endorphin—which may be raised by social music-making—has been implicated in resilience, stress, and PTSD (Bali et al., 2015) as has the neuropeptide nociceptin (Tollefson et al., 2017; Narendran et al., 2019). Nociceptin receptor polymorphisms have been linked to the severity of PTSD, but unlike β-endorphin, no relationship to music has been examined. Finally. there is also evidence of an important role for dopamine in the pathophysiology of PTSD (Lee et al., 2017; Torrisi et al., 2019) with potential interaction with oxytocinergic systems (Zhang et al., 2019), further strengthening the suggestion about the potential usefulness of music therapy given the known impact that music has on dopaminergic motivation and reward systems in the human brain (Chanda and Levitin, 2013; Zatorre and Salimpoor, 2013; Ferreri et al., 2019).

### Learning, Social Memory, and Hippocampal Plasticity

In children, some degree of music training has a significant impact on brain structure and plasticity as well as having a positive influence on social, empathic, cognitive and academic development (e.g., Schlaug et al., 2009; Kirschner and Tomasello, 2009; Schellenberg et al., 2015; Habibi et al., 2018; Sachs et al., 2018; de Manzano and Ullén, 2018; Guhn et al., 2020). Learning to play an instrument requires the recruitment of many sensorimotor systems and circuits, and many studies have reported that music training has beneficial effects on various executive functions and some types of memory, benefits that are maintained throughout a person’s lifetime and may be protective against cognitive decline (Talamini et al., 2017; Mansens et al., 2018). Once again there are several intriguing and potentially important links between music training, music-related activities, and the neuroscience of oxytocin, in this case, the links that are relevant to memory and aging, with dance and exercise adding an additional dimension to the discussion. Oxytocin’s effects on social recognition, learning, and memory are associated with activity in the hippocampus, amygdala, nucleus accumbens, and prefrontal cortex (e.g., Ferguson et al., 2002; Hurlemann et al., 2010; Mitre et al., 2016; Grinevich and Stoop, 2018; Jurek and Neumann, 2018; Lin and Hsu, 2018; Lin et al., 2018; Lopatina et al., 2018; Cilz et al., 2019; Tan et al., 2019; Raam, 2020; Xu et al., 2020). In the following discussion, the focus is primarily on the hippocampus, given its role in consolidating, integrating and retrieving personal autobiographical memories (Bonnici and Maguire, 2018; Sheldon et al., 2019). There is a huge literature on hippocampal connectivity and plasticity related to these dynamic and transformational processes—the emphasis here will be limited to several aspects of social learning and memory perhaps most relevant to a review of music and oxytocin.

Music activates diverse regions and circuits within the CNS including, depending on context and emotional valence, essentially the same limbic structures that are responsive to oxytocin (Boso et al., 2006; Koelsch, 2014, 2018). Music training and practice improves memory (Talamini et al., 2017; Mansens et al., 2018) and affects the architecture and organization of both gray and white matter in the brain (de Manzano and Ullén, 2018). Of particular relevance here is the positive effect that music training has on gray matter volume and plasticity in the hippocampus (Herdener et al., 2010), and whether this may be in some way related to increased endogenous oxytocin and reduced cortisol levels in individuals involved in musical activities—by what mechanisms could music, memory and oxytocin be linked? Acting through its receptor, oxytocin can act both pre-and postsynaptically to enhance LTP, alter the balance of excitatory and inhibitory activity, and modulate synaptic plasticity (Tomizawa et al., 2003; Lee et al., 2015; Bakos et al., 2018; Lin and Hsu, 2018; Tirko et al., 2018), These are all critical elements during the process of learning, socialization and memory consolidation (Ferguson et al., 2000; Lin et al., 2018), and intranasal application of the peptide at low doses is known to enhance social memory in human subjects (Jurek and Neumann, 2018).

### Neurogenesis

The hippocampal dentate gyrus appears to be one of the few sites in the adult mammalian CNS where new neurons are born throughout life (neurogenesis). This ongoing process is thought to be important in learning and in facilitating the addition of new memories onto similar previous experiences and knowledge, minimizing overlap in the resultant patterns of activity so that particular events can be discriminated from each other (e.g., Conçalves et al., 2016; França et al., 2017; Alam et al., 2018; Toda and Gage, 2018; Licht et al., 2020). In animals, experimental disruption of neurogenesis impairs social memory and coping with stress (Clelland et al., 2009; Garrett et al., 2015; Alam et al., 2018). Social interactions enhance new neuronal birth (Hsiao et al., 2014) whereas social isolation and stress-related changes that include increased cortisol levels lead to a reduction in neurogenesis (McEwen, 1999; Cinini et al., 2014; Opendak et al., 2016; Snyder and Drew, 2020), negatively affecting cognition, learning, and memory (Ouanes and Popp, 2019).

Oxytocin protects the hippocampus from stress-related effects including the negative impact of corticosterone treatment and directly induces neurogenesis in the adult rodent dentate gyrus (Lee et al., 2015; Sánchez-Vidaña et al., 2016; Lin et al., 2017; Lin and Hsu, 2018). The peptide also promotes the differentiation and dendritic maturation of these new neurons with associated effects on social behavior (Sánchez-Vidaña et al., 2016). This influence of oxytocin on hippocampal neurogenesis and social learning is indirectly enhanced by the peptide’s actions in increasing BDNF expression (Dayi et al., 2015; Havranek et al., 2015; Zhang et al., 2020). This neurotrophin plays a key role in hippocampal plasticity and neurogenesis (Miranda et al., 2019). Its expression in the hippocampus is negatively affected by stress (Bennett and Lagopoulos, 2014; Dayi et al., 2015) but is significantly increased by physical exercise (Ding et al., 2011). In animals, increased BDNF levels are correlated with increased neurogenesis, the greater the amount of exercise the greater the proliferation of new neurons (reviewed in Liu and Nusslock, 2018). Indeed, it was recently shown that intense physical activity releases breakdown products from the muscle that act on promoters to increase BDNF gene expression and protein (Sleiman et al., 2016; Stephan and Sleiman, 2019).

There remains some controversy as to whether new neurons are born and survive within the adult human dentate gyrus (Sorrells et al., 2018; Duque and Spector, 2019); however, the weight of evidence and opinion is that neurogenesis and neuronal turnover does occur (Spalding et al., 2013; Boldrini et al., 2018; Kempermann et al., 2018; Kuhn et al., 2018; Cope and Gould, 2019; Horgusluoglu-Moloch et al., 2019; Lima and Gomes-Leal, 2019; Petrik and Encinas, 2019; Tobin et al., 2019; Lucassen et al., 2020), although estimates of the number of neurons born each day vary, and numbers may decline with age and disease (Moreno-Jiménez et al., 2019; Snyder, 2019). It is however clear that exercise and cardiovascular fitness are correlated with increased hippocampal volume and improved cognitive function (Erickson et al., 2011; Stillman et al., 2016). Furthermore, while it is not yet known if such changes are associated with enhanced neurogenesis, hippocampal size in humans is correlated with plasma BDNF levels (Erickson et al., 2011).

It is well established that neural activity in the hippocampus and other parts of the limbic system is altered by listening to music. Given this, and what is known about the effects of music on hormones such as oxytocin and cortisol, it will be of interest to determine if participation in musical activities influences human hippocampal neurogenesis (Fukui and Toyoshima, 2008), and how this might relate to the known beneficial effects of music on memory and cognition. Such activities should include movement and dance which are entrained within the diverse neural networks responsive to music (e.g., Brown et al., 2006; Phillips-Silver and Trainor, 2007; Nozaradan et al., 2011). Dance not only increases cooperation and group synchrony (Reddish et al., 2013; Karpati et al., 2016; Chauvigné et al., 2019) but improves fitness in the elderly (Douka et al., 2019). Measurement of oxytocin, cortisol, and BDNF in dancers seems warranted, and it may well be that, in addition to potential oxytocin-mediated effects, exercise and cardiovascular fitness associated with dancing are capable of adding an important extra dimension to the social, physical and mental health benefits of music appreciation and music-related activities, perhaps especially in the elderly.

### Systemic Effects—Further Links Between Oxytocin and Musicality

In addition to its physiological effects on CNS function, oxytocin has been reported to have even broader health benefits. The peptide decreases the progression of atherosclerosis and protects against cardiovascular disease (Reiss et al., 2019; Wang et al., 2019; Buemann and Uvnäs-Moberg, 2020), in association with social engagement (Ulmer-Yaniv et al., 2016; Walker et al., 2019) it has beneficial effects on the immune system, and it lowers cytokine levels and inhibits inflammation (Li et al., 2017b; Reiss et al., 2019). Oxytocin has also been reported to regulate appetite and food intake (Lawson et al., 2019; Onaka and Takayanagi, 2019; Quintana et al., 2019). Associated with these multiple beneficial effects, the peptide has been found to lower blood pressure and assist in maintaining glucose homeostasis, potentially useful as a therapeutic tool in the treatment of type 2 diabetes and obesity (Reiss et al., 2019). Again, many of these systemic oxytocinergic effects overlap those that can be elicited by listening to and/or performing music, including a reduction in blood pressure, the modification of immune responses and inflammatory markers, reduction of anxiety and stress and a moderating effect on blood glucose levels (Koelsch and Jäncke, 2015; Finn and Fancourt, 2018; Kang et al., 2018). The potentially additional benefits of music-related exercises such as dance have already been alluded to in the preceding paragraphs.

## Conclusion and Therapeutic Implications

Given what is increasingly becoming known about the neurological and systemic effects of oxytocin, it is important to analyze further how music and dance influence this peptide and its downstream pathways, and the extent to which social musical activities drive some of the interactions between oxytocin and other neuromodulatory systems such as dopamine, BDNF, and the various endogenous opioids. From an evolutionary perspective, it may clarify the extent to which evolving musical capabilities in modern humans took advantage of the ancient oxytocinergic network to facilitate prosocial interactions, promote trust and reciprocal affiliative behaviors, and help reduce levels of anxiety and individual insecurity throughout life. It will also contribute to a better understanding of the mechanisms that underlie the mental and general health benefits of music, its remarkable emotional and mnemonic power, it’s capacity to alter brain architecture, and its ability to revitalize episodic memories (Zhang et al., 2017; Särkämö and Sihvonen, 2018), especially vulnerable in early stages of Alzheimer’s disease (Groussard et al., 2019; Slattery et al., 2019).

From a therapeutic perspective, dancing is already a useful tool in the treatment of Parkinson’s disease (Pereira et al., 2019); perhaps for some dementia sufferers, in addition to singing, dancing to favorite tunes may be even more beneficial when promoting social interactions with partners and unlocking autobiographical memories. Similarly, the use of intranasal oxytocin as a therapeutic tool in conditions such as PTSD shows promise (Giovanna et al., 2020) and may be even more effective when used with other treatments including combination with appropriate prosocial music-related activities. As another example, the combined use of music and the anti-nociceptive properties of oxytocin may enhance the therapeutic efficacy of strategies aimed at reducing the perception of chronic (Garza-Villarreal et al., 2017; Hilfiger et al., 2020; Schneider et al., 2020) or peri-operative pain (Nilsson et al., 2005, 2009; Nilsson, 2009; Van der Heijden et al., 2015). Last but not least, the use of oxytocin in the treatment of autism spectrum disorders (Yamasue and Domes, 2018) and other psychiatric conditions (Peled-Avron et al., 2020) may benefit from synergistic application with appropriate music-related therapeutic strategies (Quintin, 2019).

## Author’s Note

During revision of this manuscript, a review was published that emphasized the importance of studying the biology of oxytocin systems in animals to better understand the translational potential of this peptide in psychiatry and mental health (Grinevich and Neumann, 2020). The reader is encouraged to access this review to complement the more focussed emphasis on human musicality described herein.

## Author Contributions

The author confirms being the sole contributor of this work and has approved it for publication.

## Conflict of Interest

The author declares that the research was conducted in the absence of any commercial or financial relationships that could be construed as a potential conflict of interest.
